# Functional relationship between material property, applied frequency and ozone generation for surface dielectric barrier discharges in atmospheric air

**DOI:** 10.1038/s41598-017-06038-w

**Published:** 2017-07-25

**Authors:** Sherlie Portugal, Subrata Roy, Jenshan Lin

**Affiliations:** 1 Department of Electrical and Computer Engineering, University of Florida, Gainesville, 32611 USA; 2 Department of Mechanical and Aerospace Engineering, University of Florida, 32611 Gainesville, USA; 3 School of Electrical Engineering, Technological University of Panama, Panama City, Panama

## Abstract

We report the experimental characterization of ozone generation in dielectric barrier discharges as a function of the material and characteristics of the dielectric barrier, operating frequency and the power consumed by a surface DBD-plasma reactor in air at atmospheric pressure. To identify the effect of the dielectric barrier, ozone production curves corresponding to ten dielectric barriers with different effective thicknesses and thermal properties are compared and analyzed for two combinations of voltage amplitudes and frequencies: 7 kV/10 kHz and 8.5 kV/14 kHz. The influence of the operating frequency over the ozone generated by a DBD-plasma reactor is studied by varying the frequency in the range 8–20 kHz. The correlation between power measurements and ozone concentrations as well as ozone quenching effects at extreme power conditions are also discussed.

## Introduction

The improvement of scientific instruments and numerical models has propelled the discovery of new applications for surface dielectric barrier discharge (DBD) plasma in recent years. Among these applications, flow control and sterilization of pathogens in air volumes and body surfaces are of great interest due to their promising benefits in aerospace, transportation, healthcare, food industry, military, and space exploration, among others.

Specifically in healthcare, total bacterial annihilation has been achieved by exposing contaminated objects directly to the DBD-plasma or by placing them in proximity to the DBD-plasma reactor in a closed chamber^1, 2^. Although early studies conducted in air at low pressure pointed to UV-C photons (100–280 nm wavelength), released by the reactions of *N*_*2*_, *O*_*2*_ and *NOH* molecules, as the main agents of bacterial annihilation^1^, recent experiments in air at atmospheric pressure did not detect UV-C among the UV radiation produced by dielectric barrier discharges^2, 3^. Furthermore, it was demonstrated that, under such conditions, ozone (*O*_*3*_) generated from plasma reactions plays the major role in bacterial killing^2^.

While published literature on sterilization mechanisms of ozone and other plasma products keeps expanding, the functional relationship between physical characteristics of a surface DBD-plasma reactor and ozone production is yet to be properly documented. Recent developments on DBD-plasma reactors originate from studies associated with flow control applications; where physical design parameters, such as arrangement of the electrodes and characteristics of the dielectric barrier, have been considered based on their influence to control flow separation and drag. However, to apply DBD based sterilization concepts in real world environments it is essential to isolate and understand the behavior of ozone as a function of key parameters that control dielectric barrier discharges, such as frequency, applied voltage and physical design of the plasma reactor. This information is crucial to develop compact and efficient electronic circuitry working in synchrony with the plasma reactor. In this regard, this paper aims to determine and report the degree to which some of these factors affect ozone generation, giving special attention to the influence of the dielectric barrier.

Although variations in ozone levels were observed when two DBD-plasma reactors with different dielectric materials were operated under the same conditions of voltage and frequency^4^, these effects have not been studied or quantified extensively. Therefore, the influence of the dielectric barrier on ozone yield is studied in this paper by conducting experimental tests on 10 different dielectric materials with different electrothermal properties and chemical composition. In addition, a second set of experiments is carried out to report the formation of ozone versus the operating frequency. For this purpose, a dielectric barrier is subjected to an alternate voltage whose frequency is varied in the range 8–20 kHz. Finally, the influence of extreme conditions of dielectric power dissipation on ozone generation is also analyzed and discussed.

## Physical configuration of the DBD-plasma reactor

**Figure 1:**
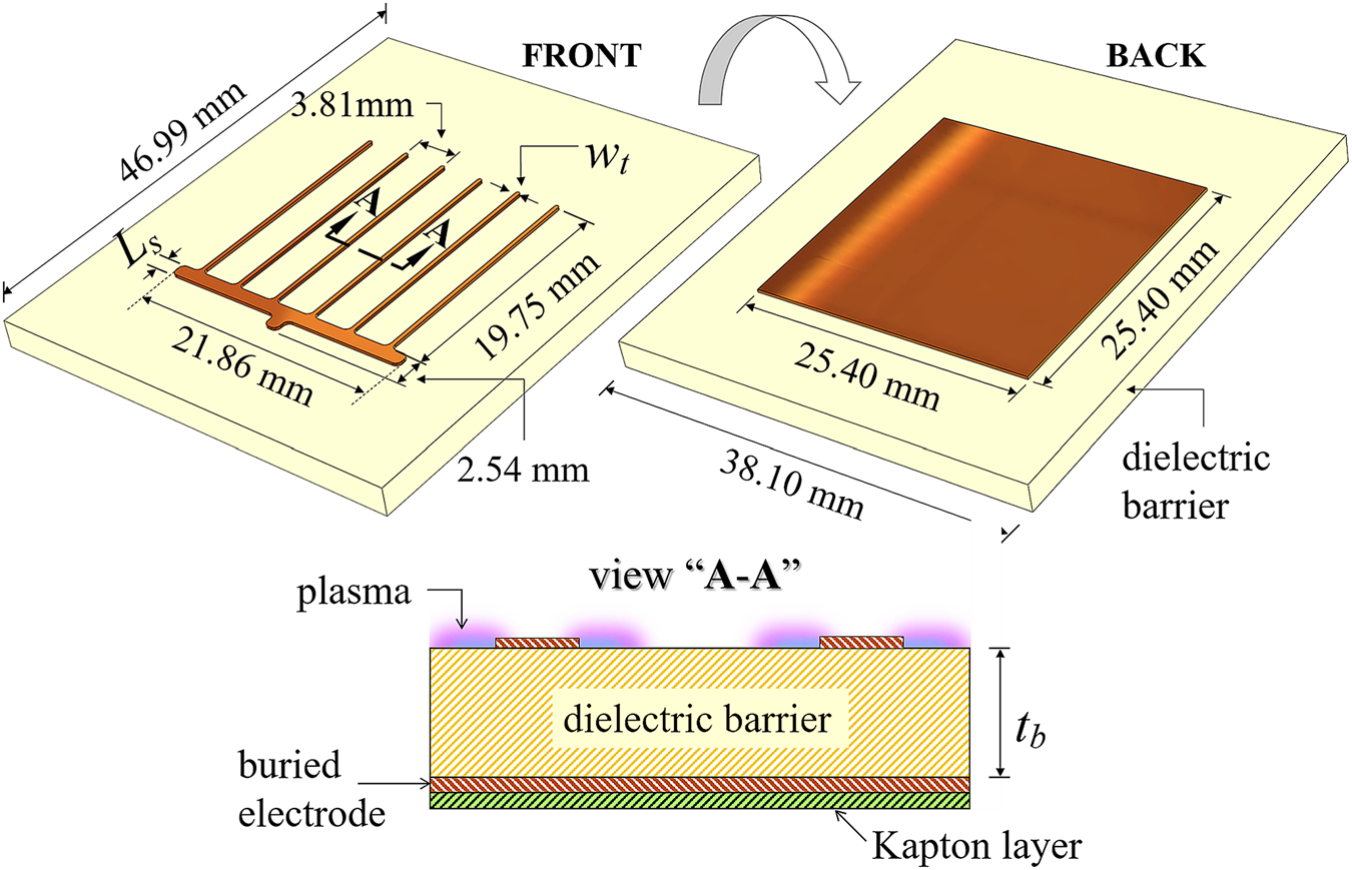
Configuration of the comb shaped DBD reactor. The exposed and buried electrodes are located on the front and back of the reactor, respectively. A cross-sectional view “A-A” shows the formation of plasma on the surface of the dielectric barrier around the edges of the exposed electrode. *w*_*t*_ = width of each teeth (0.38 mm); *L*_*s*_ = length of the shaft (1.27 mm).

In its most basic form, a surface DBD-plasma reactor consists of two electrodes asymmetrically positioned on both sides of a dielectric (barrier) material. One of the electrodes is exposed to the surrounding gas whereas the second one is either buried in the dielectric barrier or covered by a layer of dielectric material, such as Kapton tape, to prevent discharge from both sides of the reactor. The specific configuration of the reactor fabricated for this study, shown in Fig. 1, is similar to that reported earlier by our group^2, 4^. Here, the exposed electrode located at the front of the reactor consists of a comb-like structure, where the shaft has a width and length of 22.86 mm and 1.27 mm, respectively. Each of the six teeth perpendicular to the shaft is 19.75 mm long and 0.38 mm wide. The exposed electrode was carefully designed to avoid areas of excessive electric flux density, such as sharp corners that could cause hot-spots and unnecessary increase in power consumption. The electrode at the back of the reactor is a square with area of 25.4 mm × 25.4 mm and it was insulated with a layer of Kapton tape of 0.09 mm. Both electrodes are separated by a dielectric barrier with an overall area of 46.99 mm × 38.1 mm.

The generation of DBD plasma begins by applying a high alternating potential difference (kV range) between the two electrodes. Charge buildup on the surface of the dielectric material above the buried electrode induces a local electric field between the exposed electrode and the barrier. When this local field exceeds the breakdown voltage of the air above the barrier, electron avalanche mechanisms give rise to the formation of weakly ionized plasma channels (streamers) and flow of electron current. These microdischarges end when the local field collapses due to the charge transfer and accumulation on the barrier’s surface. Otherwise the microdischarges would develop into thermal arcs. Figure 1 also illustrates the operation of the comb shaped reactor. A zoomed cross-sectional view “A-A”, added for clarity, shows the formation of plasma over the surface of the dielectric around the edge of the exposed electrode. In this configuration, the plasma extends along the entire perimeter of the exposed electrode; therefore, the total length of the plasma envelope is approximately 281.59 mm.

Each tooth of the comb reactor has a capacitance between the exposed and ground electrodes given by: *C*_*a*_ = (*ε*_0_ *ε*_*r*_ *A*_*e*_)/*tb*, where *ε*_0_ is the permittivity of vacuum (8.854 × 10^−12^ *F*/*m*), *ε*_*r*_ is the relative permittivity of the dielectric material, *A*_*e*_ is the area of the exposed electrode, and *t*_*b*_ is the thickness of the dielectric barrier. Hence, the surface charge density on the exposed electrode can be expressed as *ρ*_*S*_ = *ε*_0_ *V*_*a*_/*t*_*eff*_, where *V*_*a*_ is the applied potential-difference between the plates and *t*_*eff*_ = *t*_*b*_/*ε*_*r*_ is the effective thickness of the dielectric.

**Figure 2:**
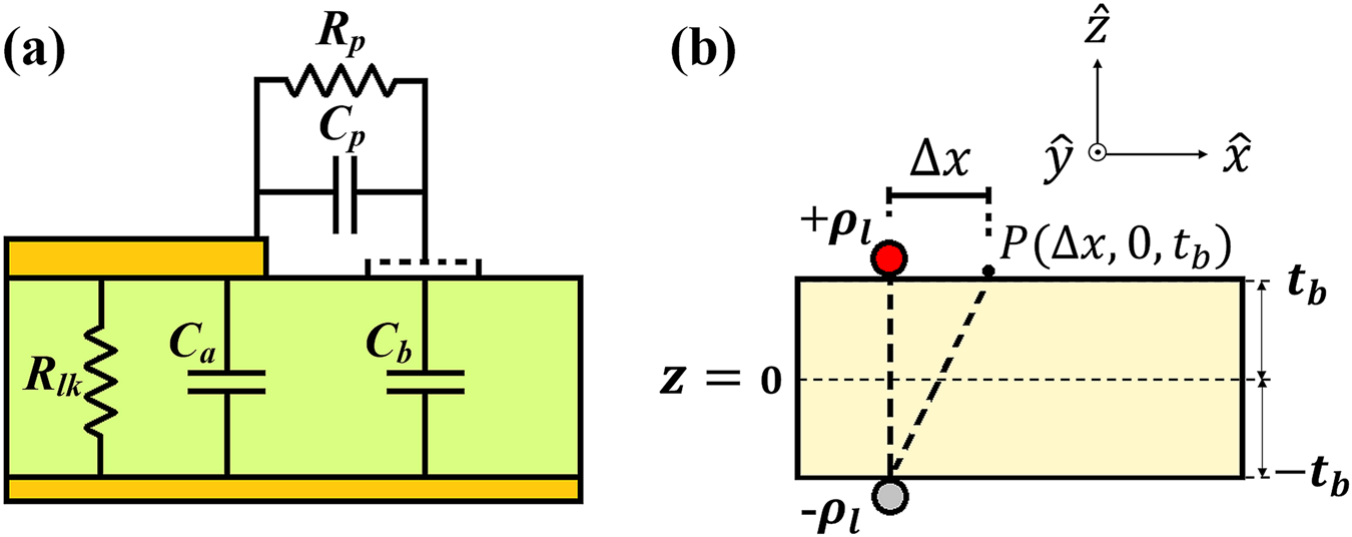
Electrical model of the DBD reactor cross-section. (**a**) Equivalent circuit of each tooth of the comb structure in the DBD reactor. (**b**) Diagram used to calculate the surface potential using method of images.

When the level of applied excitation is enough to induce DBD-plasma formation, the plasma reactor can be modeled according to Fig. 2a ^5, 6^. Here, a capacitance *C*_*p*_ is formed between the edge of the exposed electrode and a virtual electrode induced by charge accumulation on the surface of the dielectric barrier. The power dissipated in the plasma is represented as a resistive element *R*_*p*_, whose resistance value oscillates between a few ohms (plasma on) and near-infinite values (plasma off). *C*_*b*_ is the barrier’s capacitance between the ground electrode and the virtual electrode. The leakage resistance *R*_*lk*_ in parallel with *C*_*a*_ represent the losses in the dielectric. Thus, the resulting impedance of this circuit is given by: *Z* = $({R}_{lk}{X}_{eq}^{2}-j{R}_{lk}^{2}{X}_{eq})/({R}_{lk}^{2}+{X}_{eq}^{2}),$ where *X*_*eq*_ = 1/*ωC*_*eq*_ is the reactance of the equivalent capacitance *C*_*eq*_ = *C*_*a*_ + *C*_*p*_ *C*_*b*_/(*C*_*p*_ + *C*_*b*_) and *ω* = 2*πf* is the angular frequency. Since for the dielectric barrier *R*_*lk*_ is very large, the reactance is the dominant term and the impedance is simplified to *Z* ≈ 1/*jωC*_*eq*_. The charge along the edge of the exposed electrode can be considered as a line charge *ρ*_*l*_ as illustrated in Fig. 2b. Through image theory, it is possible to calculate the potential Φ_*b*_ at a point *P* on the dielectric surface very close to the edge of the exposed electrode. Considering Δ*x* << 2*t*_*b*_, approximates the potential of a line charge over a conducting plane, shown in equation (1)^7, 8^.

1 $${V}_{b}={{\rm{\Phi }}}_{b}=\frac{{\rho }_{l}}{2\pi {\varepsilon }_{o}}\,\mathrm{ln}(\frac{2{t}_{b}}{{\rm{\Delta }}x})$$

These simplified calculations serve to give a projection of the dependency of the dielectric barrier and DBD-plasma formation. Since, the potential difference between exposed electrode and barrier’s surface is given by *V*_*p*_ = *V*_*a*_ − *V*_*b*_, we can expect an inverse proportionality between *V*_*p*_ and the logarithm of the barrier’s thickness. However, to understand the influence of the characteristics of different dielectric materials we must consider the relative permittivity of the material. Therefore, in this study the influence of the dielectric material of the barrier is studied through the parameter *t*_*eff*_ since it encompasses the effects of both *t*_*b*_ and *ε*_*r*_.

## Experimental Procedure

### Dielectric materials

The dielectric materials used in this experimental study are listed in Table 1. These materials differ in physical properties and chemical composition. Their thicknesses and dielectric constants were selected to cover a reasonable range of *t*_*eff*_. Included are ceramics such as *Al*_*3*_ *O*_*2*_, polymers such as *PTFE*, and a variety of composites. Other materials like quartz, commonly used in biomedical applications, will be consider it in future studies.

**Table 1:** Main properties of the materials used as dielectric barrier in the DBD plasma reactor.

No.	*t*_*eff*_	*t*_*b*_	Trade name	Chemical description	*ε*_*r*_	tan*δ*	MA (%)
1	0.03	0.10	Ultralam 3850HT	liquid crystalline polymer	3.14	0.0020	0.04
2	0.05	0.64	RT/6010LM	ceramic-PTFE	10.97	0.0023	0.01
3	0.07	0.64	curamik®	Alumina (Al_2_O_3_) 96%	9.0	0.0002	0.00
4	0.09	0.64	RO3006	ceramic-PTFE	6.54	0.0020	0.02
5	0.11	1.00	curamik®	Alumina (Al_2_O_3_) 96%	9.0	0.0002	0.00
6	0.20	0.76	RO4350B	hydrocarbon/ceramic	3.48	0.0031	0.06
7	0.36	0.76	Teflon^TM^	PTFE	2.1	0.0003	0.02
8	0.41	1.52	RO4350B	hydrocarbon/ceramic	3.48	0.0031	0.06
9	0.50	1.52	RO3003	ceramic-PTFE	3.0	0.0010	0.04
10	0.76	1.60	Teflon^TM^	PTFE	2.1	0.0003	0.02

*t*_*eff*_ = dielectric effective thickness in millimeters; *t*_*b*_ = physical thickness of the dielectric barrier in millimeters; *ε*_*r*_ = relative permittivity of the material; tan*δ* = loss tangent or dissipation factor; MA = moisture absorption; *PTFE* = Polytetrafluoroethylene.

## Experimental setup

**Figure 3:**
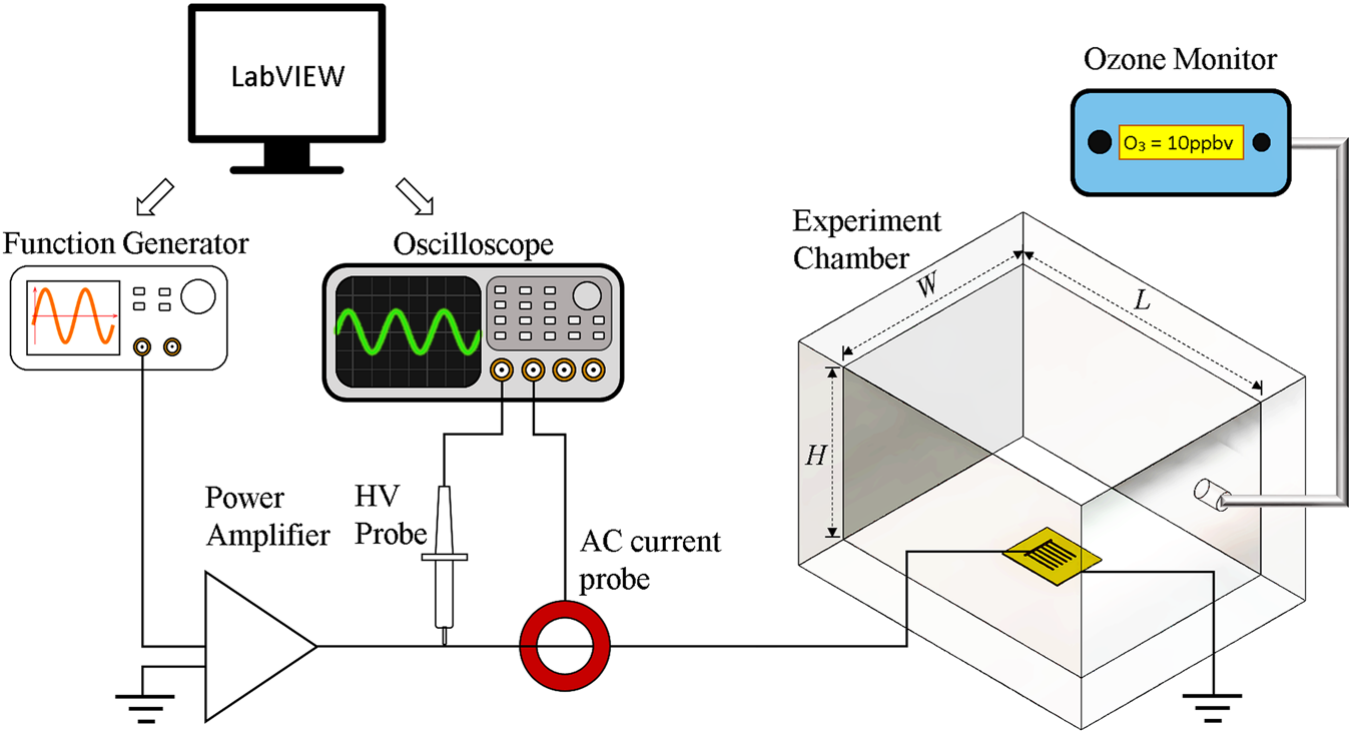
Experimental setup. *H*, *W* and *L* represent the chamber’s high (14.605 cm), width (17.145 cm) and length (22.225 cm), respectively.

An illustration of the experimental setup is shown in Fig. 3. The DBD-plasma reactor was placed inside an acrylic chamber of length *L* = 22.225 cm, width *W* = 17.145 cm and height *H* = 14.605 cm. A PTFE tubing of external/internal diameter of 6.35/3.175 mm is fitted to an opening of the chamber to provide air samples to an ozone monitor (2B Technologies model 202). This monitor reads ozone levels based on the method of UV absorbance at 254 nm and has a measurement range of 0–100,000 ppb with an accuracy of ±2% of the reading. The comb reactor was positioned at the center of the chamber with its teeth pointing towards the tube opening so that the maximum three-dimensional flow is in the direction of the ozone monitor tubing. The plasma reactor requires an alternating high voltage (kV) source. To generate such excitation level, a small voltage signal (mVpp range), whose frequency and magnitude are controlled by a function generator (Tektronix AFG3022B), is passed through a power amplifier (Crown CDi 4000) followed by a transformer. The optimal frequency range of the transformer is limited to 8 kHz–24 kHz. The actual voltage and current in the reactor are monitored with a high voltage probe (Tektronix P6015A) and an AC current probe (Pearson Electronics 2100), both connected to an oscilloscope (Tektronix DPO 3014) with sample rate of 2.5 GS/s and recording length set to 1 million points. The power dissipated in the plasma is calculated by numerically integrating the product of the voltage and current waveforms. A LabVIEW code was developed to control the function generator and the transfer of voltage and current data to a computer every ten seconds, the same interval of the ozone readings. Temperature and humidity inside the chamber were monitored with a humidity and temperature chart recorder (EXTECH Instruments RH520A-NIST). The initial conditions for each experiment were set at 23 °C and a relative humidity of 65%. The ozone and power data has been deposited in the figshare public repository (https://doi.org/10.6084/m9.figshare.5183452).

## Results and Discussion

### Ozone levels versus time

**Figure 4:**
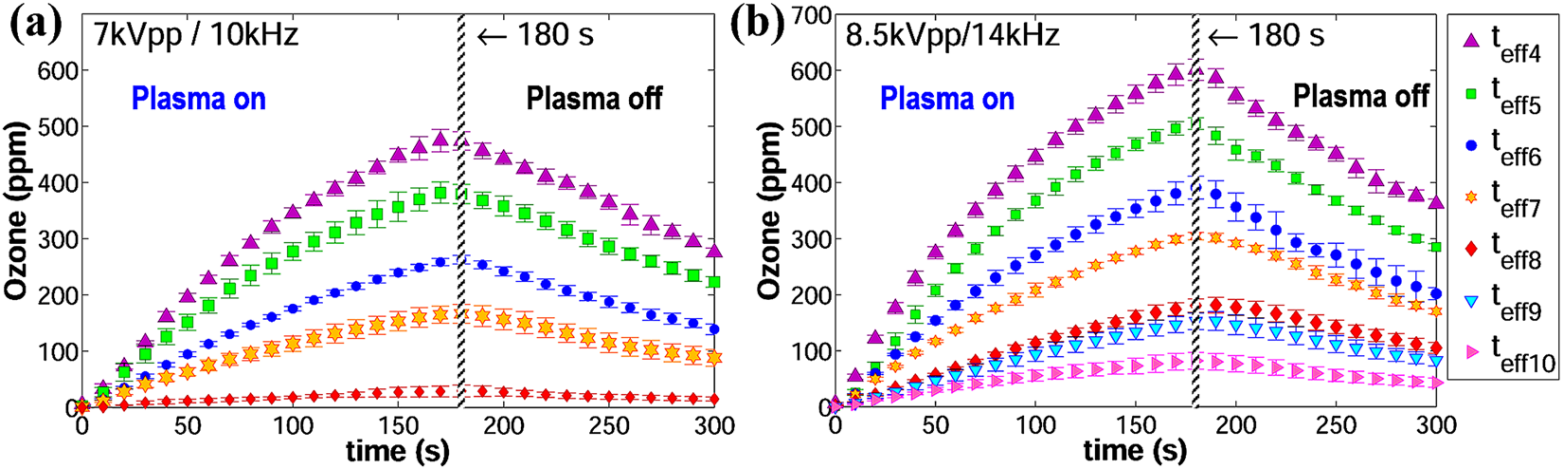
Curves of ozone vs. time corresponding to different values of *t*_*eff*_. (**a**) Voltage/frequency: 7 kVpp/10 kHz. (**b**) Voltage/frequency: 8.5 kVpp/14 kHz.

The ozone concentrations as a function of time corresponding to the voltage/frequency pairs of 7 kVpp/10 kHz and 8.5 kVpp/14 kHz are illustrated in Fig. 4a,b, respectively. The selection of voltage/frequency requires compromise because the applied AC voltage must be high enough to produce sufficient plasma on the reactors with the largest effective thicknesses, but low enough to avoid exceeding the breakdown threshold of the thinnest barriers. Thus, the selected voltage/frequency of 7 kVpp/10 kHz was safe enough to power the plasma reactors with the smallest values of *t*_*eff*_ without breaking them, but the resulting power did not generate sufficient plasma at *t*_*eff*__9_ and *t*_*eff*_10. On the contrary, 8.5 kVpp/14 kHz generated reliable amounts of plasma at *t*_*eff*__9_ and *t*_*eff*__10_, but damaged the reactor with *t*_*eff*__1_.

The ascending part of the curves (DBD-plasma on) in Fig. 4 can be approximated to the quadratic equation *O*_3_(*t*) = −*at* ^2^ + *bt* ± *c*, where *a* is the quadratic coefficient that determines the opening of the parabola, *b* is the linear coefficient that shifts the vertex position and *c* is the intersection point with the *O*_*3*_-axis that can be approximated to 0. After three minutes, the plasma reactor was powered off and the ozone curves follow an exponential decay *O*_3_(*t*) = *A*exp(−0.005 *t*), where *A* is the ozone level immediately after the reactor is turned off. Since these equations are related to the specific volume of the chamber, they will not be used in the analysis of the experimental results.

Curves corresponding to *t*_*eff*__1_, *t*_*eff*__2_, *t*_*eff*__3_ in Fig. 4a and *t*_*eff*__2_, *teff*_*eff*__3_ in Fig. 4b are absent because for these thin dielectrics the applied voltage/frequency yielded power levels close to the maximum capacity of the reactors. These cases are discussed separately in subsection 4.5, since they exhibit a different behavior related to excess power dissipation.

The error bars in Fig. 4 represent the total uncertainty in a 95% confidence interval. The total uncertainty includes the random error (standard error of the mean) and the systematic error introduced by the accuracy of the ozone monitor (±2%).

### Ozone rate analysis

**Figure 5:**
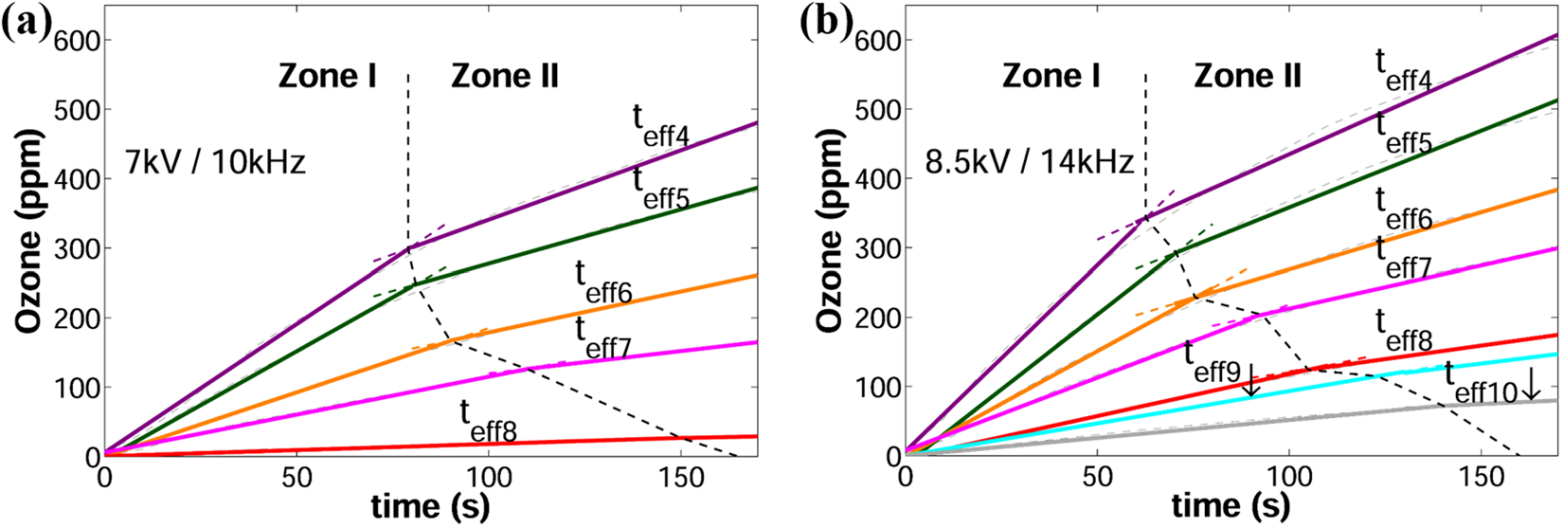
Slopes of ozone production curves corresponding to different values of *t*_*eff*_. (**a**) Voltage/frequency: 7 kVpp/10 kHz. (**b**) Voltage/frequency: 8.5 kVpp/14 kHz.

The slopes of ozone curves for the voltage/frequency pairs of 7 kVpp/10 kHz and 8.5 kVpp/14 kHz are presented in Fig. 5a,b, respectively. In both cases, the analysis of the ozone rate can be separated into two regions. The first region, designated as *zone I*, starts at very low ozone concentrations and is characterized by the linear growth of ozone levels. In this zone, the bombardment of electrons give rise to very fast dissociation processes of *O*_*2*_ and *NO* molecules to produce oxygen atoms. This triggers the rapid formation of ozone through the chemical reaction: $[O+{O}_{2}+M\to {O}_{3}^{\ast }+M\to {O}_{3}+M],$ where *M* is a third collision partner (*O*,*O*_2_,*O*_3_,*N*_2_) and ${O}_{3}^{\ast }$ is an excited ozone molecule. Additional chemical reactions involving nitrogen, such as [*N* + *O*_2_ → *NO* + *O*], [*N* + *NO* → *N*_2_ + *O*], $[{N}_{2}(A\,{}^{3}{\rm{\Sigma }})+{O}_{2}\to {N}_{2}O+O],$ and $[{N}_{2}+{O}_{2}(A\,{}^{3}{\rm{\Sigma }})\to {N}_{2}+2O]$ also contribute with oxygen atoms and enhance the ozone formation^9, 10^. The second region, designated as *zone II*, starts when the initial ozone rate decreases due ozone dissociation processes and no longer follows a linear trend. As concentrations of both ozone molecules and oxygen atoms increase, chemical reactions that either consume ozone molecules [*O* + *O*_3_ + *M* → 2*O*_2_ + *M*], $[O+{O}_{3}^{\ast }+M\to 2{O}_{2}+M]$ or compete with their formation [*O* + *O* + *M* → *O*_2_ + *M*] become more frequent and eventually lead to steady ozone levels (zero slope), because the amount of ozone created is within the same order of magnitude as the ozone being destroyed^11^.

In this work, the time boundary between *zone I* and *zone II* was established as the point where the ozone curve deviates more than 5% from the linear slope of *zone I*. These interpolated values are shown in Table 2.

**Table 2:** Time boundary between *zone I* and *zone II*.

*t*_*eff*_	7 kVpp/10 kHz time (s)	8.5 kVpp/14 kHz time (s)
4	79	62
5	81	72
6	90	75
7	110	93
8	150	105
9		123
10		140

### Ozone rate versus effective thickness

**Figure 6:**
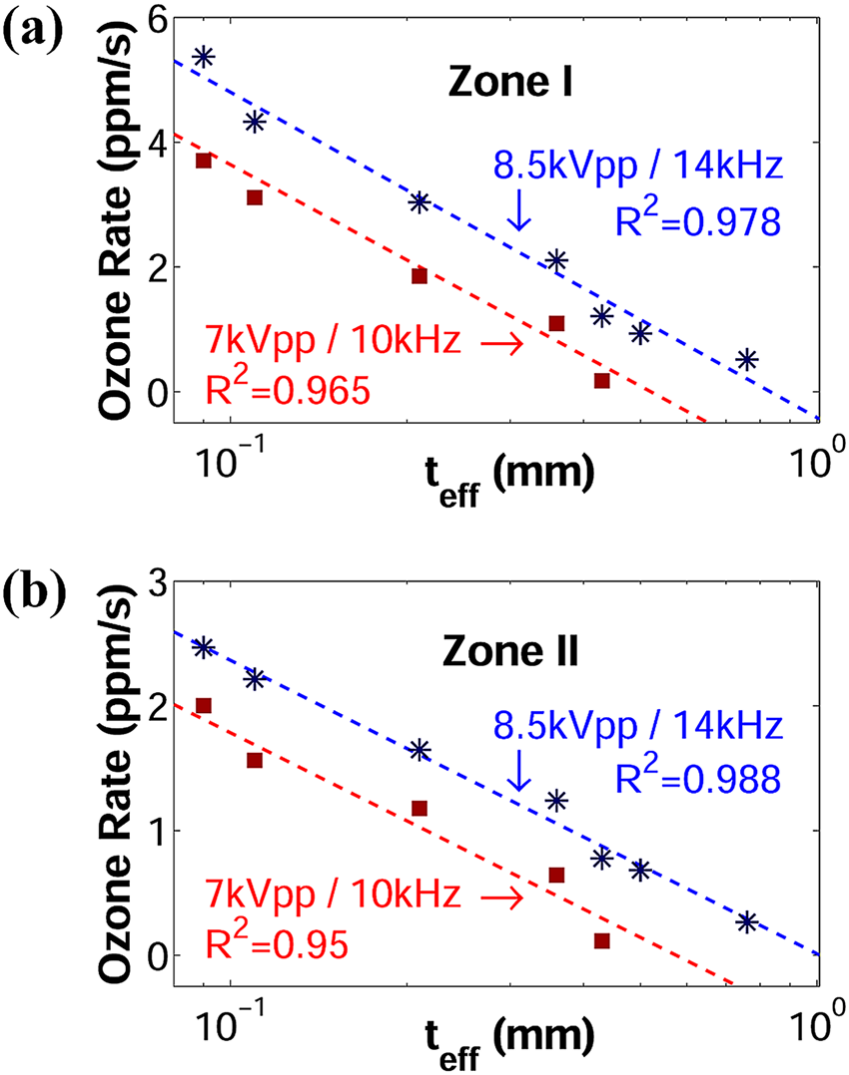
Ozone rate vs. *t*_*eff*_ at voltage/frequency pairs 7 kVpp/10 kHz and 8.5 kVpp/14 kHz. (**a**) *Zone I*. (**b**) *Zone II*.

Experimental results, shown in Fig. 6, indicate that ozone rate is inversely proportional to the logarithm of *t*_*eff*_. The statistical R^2^ value is shown for each curve. This relationship, expressed in equation (2), is consistent throughout both zones and both cases of voltage/frequency and demonstrates that ozone generation is dependent on the effective dielectric thickness of the DBD-plasma reactor and not directly on its real physical thickness.

2 $$\frac{d{O}_{3}(t)}{dt}\propto \frac{1}{\mathrm{ln}({t}_{eff})}$$

### Power as a function of the effective thickness

**Figure 7:**
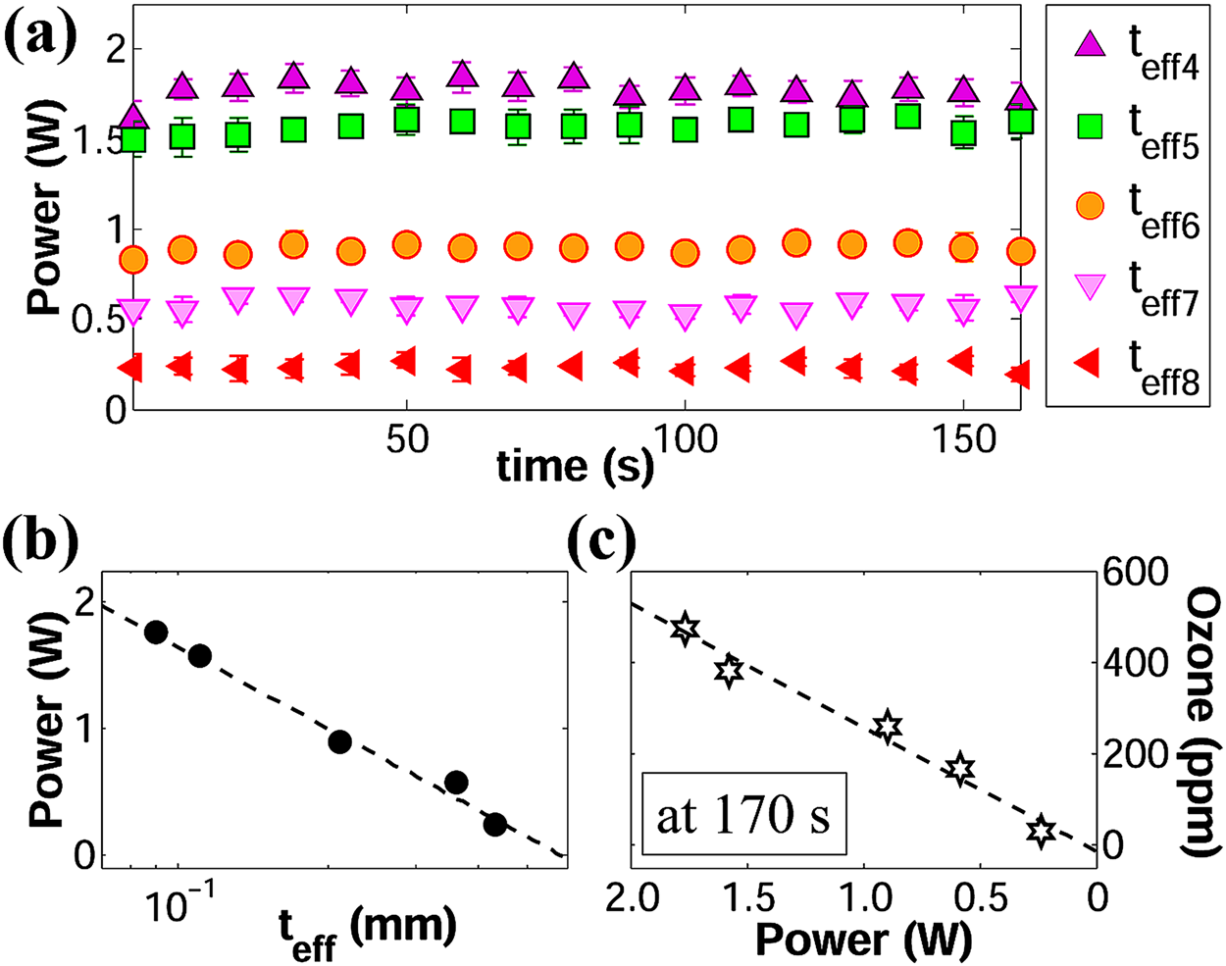
Power consumption of DBD-plasma reactors at 7 kVpp/10 kHz. (**a**) Instant power corresponding to different values of *t*_*eff*_. (**b**) Average power vs. *t*_*eff*_. (**c**) Ozone levels vs. average power at 170 s.

The power consumed by the DBD-plasma reactors remained at constant levels throughout the entire period that the plasma was on. As shown in Fig. 7a, for the voltage/frequency case of 7 kVpp/10 kHz. This behavior was consistent throughout all the experiments carried out in this study, which proves that the decline of ozone rate/levels in *zone II* occurs due to chemical reactions involving ozone molecules and not because of power fluctuations. Figure 7b shows that power is also inversely proportional to the logarithm of the effective thickness, which is expressed in equation (3).

3 $$P\propto \frac{1}{\mathrm{ln}({t}_{eff})}$$

This behavior is related to memory effects characteristic of DBD plasmas that could be explained as follows: When a microdischarge ends, the local electric field at that specific location collapses and charge accumulation on the surface of the dielectric barrier prevents the reignition of the same discharge until the next half cycle when the applied voltage changes polarity^12, 13^. In surface DBD, if the voltage in the discharge space *V*_*p*_ keeps rising, more microdischarges are produced at new locations along the edge of the exposed electrode and the discharge area is enlarged, increasing the average power consumed by the plasma. Therefore, if *V*_*p*_ decreases with the logarithm of the barrier’s effective thickness, it would be natural for the average power to follow the same trend. In addition, the fact that both ozone and power share the same proportionality with *t*_*eff*_ is an indication that instantaneous ozone production is linearly correlated with power. This latter statement is demonstrated in Fig. 7c.

The results presented above correspond to quasi-uniform and moderate-filamentary modes of DBD-plasma. Recent studies have demonstrated that on these operating conditions, power losses in the dielectric are negligible and that most of the heat in the reactor’s barrier is transferred from the plasma through convection/radiation processes^14, 15^. The materials used in this study have loss tangent (tan*δ*) values in the range 0.0002–0.0031. Loss tangent is defined as the ratio of the complex and real part of the permittivity of the material:tan*δ* = *ε*′/*ε*″ = *σ*/*ωε*_*r*_ *ε*_0_, where *σ* represents the material’s conductivity. In practice, materials having tan*δ* << 1 are considered to approach ideal dielectrics and the contributions due to *σ* maybe ignored^16, 17^. Monitoring of the temperature inside the chamber showed that during three minutes of operation of the reactor, the overall temperature increment in the chamber was less than 1 °C.

### Effects of excess power dissipation

**Figure 8:**
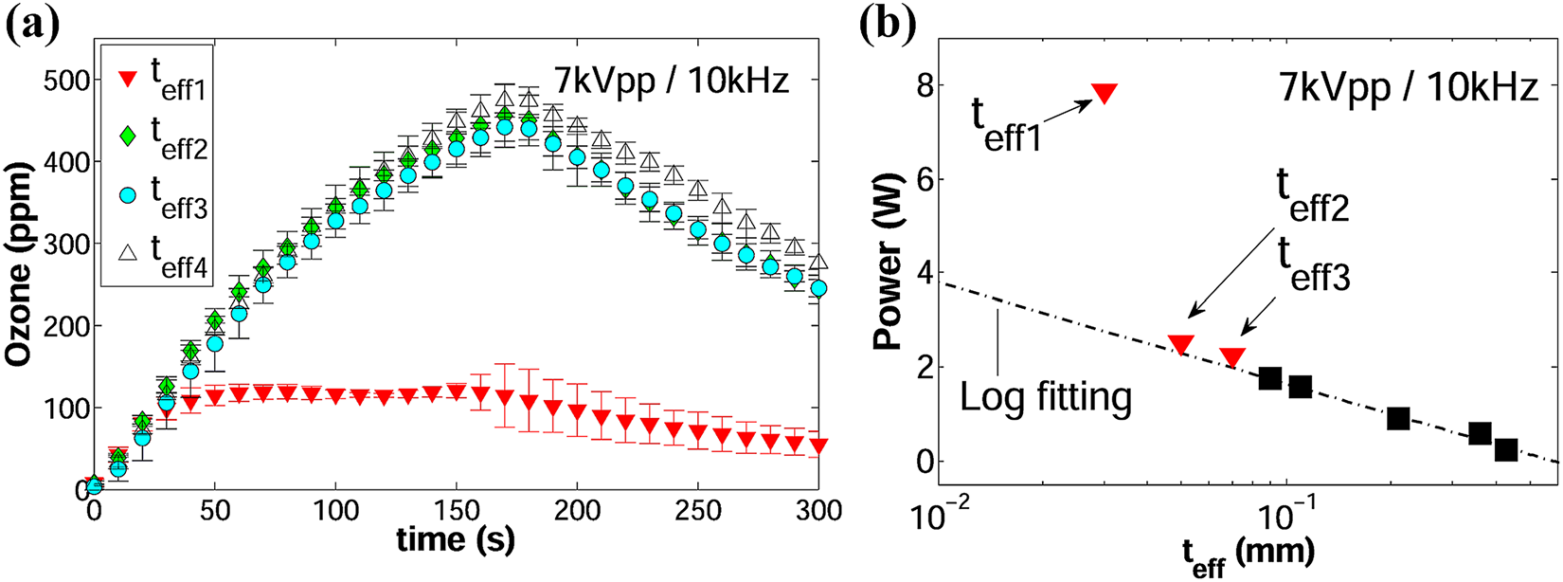
Experimental results corresponding to *t*_*eff*1_, *t*_*eff*2_ and *t*_*eff*3_ at 7 kVpp/10 kHz; *t*_*eff*4_ is only added for reference. (**a**) Ozone levels vs. time. (**b**) Average power vs.*t*_*eff*_; black square markers represent the range *t*_*eff*4_(left) to *t*_*eff*8_(right).

At atmospheric pressure, the surface DBD-plasma has a filamentary morphology. At low power levels (highest values of *t*_*eff*_), the plasma exhibits a quasi-uniform mode characterized by glow discharge spots during the negative cycle and streamer discharges during the positive cycle^14^. As power increases with the reduction of *t*_*eff*_ defined filaments start to form. These filaments are also referred to as leaders^13, 18^ in the literature. They are characterized by prominent brightness and elongation, and high temperature compared to the surrounding plasma. Initial levels of filamentation enhance ionization and ozone formation, such as in the case of *t*_*eff*__4_ and *t*_*eff*__5_. However, for thinner barriers, some of the filaments can induce high current peaks and significantly increase the power consumption. Under these extreme conditions *P* ∝ 1/log(*t*_*eff*_) no longer holds. Instead, ozone concentrations reach a maximum point or plateau for a short period and then follow a rapid decline, which indicates rapid quenching of ozone. This behavior is depicted in Fig. 8a for the cases of *t*_*eff*1_, *t*_*eff*2_ and *t*_*eff*3_ at 7 kVpp/10 kHz, where the curve corresponding to *t*_*eff*4_ was included as a reference. Initially, the reactors generated the same ozone levels at the same rate, suggesting a maximum rate of formation of oxygen atoms in the air volume. Soon after, these levels of ozone start to decline in a manner directly correlated to the power dissipated in the plasma, shown in Fig. 8b. The black square markers correspond to power levels in the range *t*_*eff*4_ to *t*_*eff*8_ that follow the relationship in equation (3). Starting from *t*_*eff*3_, the power levels begin to separate from the logarithmic trend, represented by a dash-dot line, and the drop of ozone levels become more critical as the power deviates from it. In addition, the overall temperature inside the chamber increased by an average of 1 °C for cases of *t*_*eff*2_ and *t*_*eff*3_, and approximately by 3 °C for the case of *t*_*eff*1_.

Ozone quenching occurs when the concentrations of *NO* and *NO*_*2*_ increase beyond a certain threshold^9, 10, 19^. In air, even very small concentrations of these molecules can induce rapid ozone depletion, because their chemical reactions consume oxygen atoms at a much faster rate than ozone reactions and destroy existing *O*_*3*_ molecules such as in the case of [*NO* + *O*_3_ → *NO*_2_ + *O*_2_]. While *NO* concentrations are mostly induced by plasma ionization processes, *NO*_*2*_ is mainly generated via the reactions between *NO* and oxygen-containing species^19^. The formation of these *NO*_*x*_ species requires much higher temperatures than the average temperature of the gas in dielectric barrier discharges. Therefore, their production must be associated to thermal filaments or leaders, which are characterized by high plasma density, high temperatures and high ionization.

### Influence of the operating frequency

**Figure 9:**
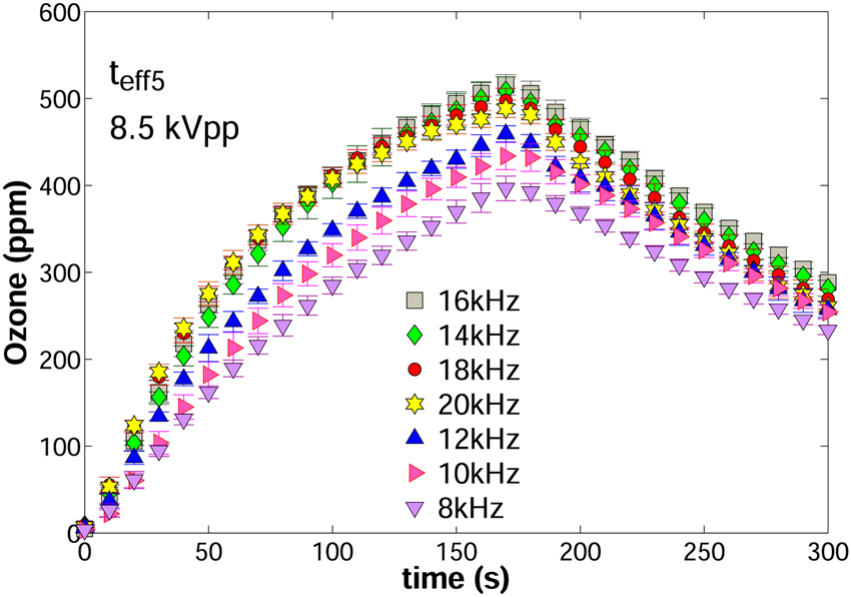
Curves of ozone vs. time in the frequency range 8–20 kHz. Applied voltage = 8.5 kVpp; *t*_*eff*_ = 0.11 mm.

The second part of this study explores the behavior of ozone as a function of the frequency of the applied voltage and determines the factors that yield such behavior. Figure 9 illustrates ozone curves corresponding to operating frequencies ranging from 8 kHz to 20 kHz. The applied voltage and *t*_*eff*_ were kept at 8.5 kVpp and 0.11 mm (*t*_*eff*5_), respectively.

$${t}_{eff}=0.11mm({t}_{eff5})$$

**Figure 10:**
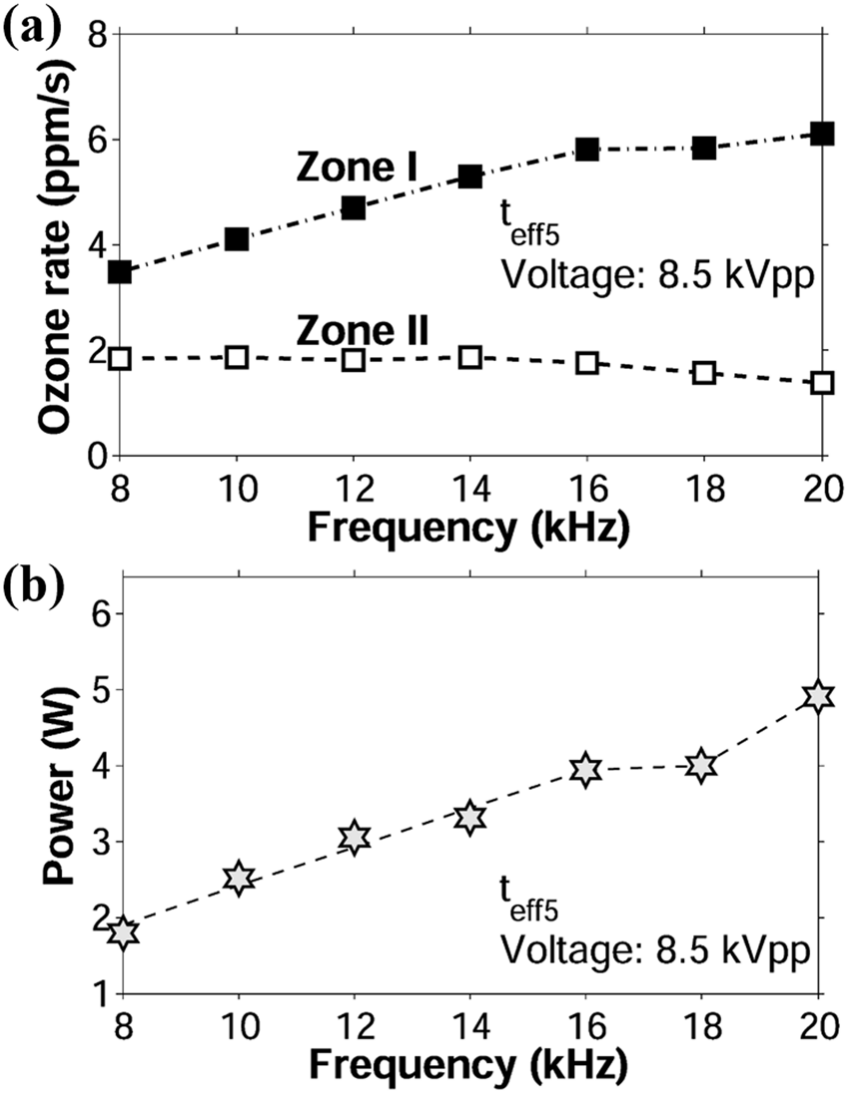
Frequency response of ozone rate and power in the frequency range 8–20 kHz. (**a**) Ozone rate vs. frequency. (**b**) Power vs. frequency. Applied voltage = 8.5 kVpp; *t*_*eff*_ = 0.11 mm (*t*_*eff*5_).

Interestingly in all curves, the transition from *zone I* to *zone II* occurred between 50 and 60 seconds. Ozone rates in both zones are plotted against frequency in Fig. 10a. These results show that from 8 to 16 kHz, the ozone rate in *zone I* increases linearly with frequency; that is,

4 $$\frac{d{O}_{3}(t)}{dt}=mf\pm c$$

where *m* is the slope of the curve, *f* is the frequency of the applied voltage and *c* is the intersection point with the *O*_*3*_-axis. The value of *m* was estimated at 0.3 ppm. In *zone II* and for the same frequency range, the ozone rate decreased due to the chemical reactions discussed previously, but it became approximately constant for all five frequencies (*m* ≈ 0). Therefore, the frequency response of the ozone rate in *zone II* can be expressed as:

5 $$\frac{d{O}_{3}(t)}{dt}=k$$

where *k* is a constant, whose value was estimated at 1.8 ppm/s for the specific case of Fig. 10a.

On the other hand, the instantaneous ozone levels in the range 8 to 16 kHz maintained a linear growth with frequency, i.e.:

6 $${O}_{3}(t)={m}_{i}\,f\pm c$$

where *m*_*i*_ is the slope of the instantaneous values of ozone versus frequency. *m*_*i*_ was calculated for all the time samples of *zone II*; its value remained relatively constant with values oscillating between 15.7 and 17.1 ppm/Hz and an average of 16.5 ppm/Hz.

Figure 10b shows that power also follows a linear increment with frequency, which is consistent with previous experimental studies^20, 21^. Power increases with frequency due to memory effects on the dielectric surface. The energy of individual microdischarges, dictated by Paschen’s Law, is independent of the operating frequency and applied voltage. However, microdischarges occur always about the same locations every half cycle. Therefore, higher operating frequencies yield more microdischarges per unit time, which translates into more power dissipation and higher levels of ionization that enhance ozone reactions.

After 16 kHz, the formation of very bright and defined plasma filaments yielded abnormally high current peaks and increased the power consumption. Ozone production corresponding to these frequencies reached a maximum initial rate (plateau) and then dropped below values corresponding to lower frequencies. Thermal filaments are known to form when the applied voltage surpasses a threshold voltage^18^. Increasing frequency reduces this threshold voltage due to the “memory voltage” effect induced by remaining charges on the dielectric barrier. The memory voltage, responsible for the self-termination of the discharge, acts in favor of the applied voltage in the following half cycle^12^. At higher frequencies, the surface charges on the dielectric barrier have less relaxation time per cycle^22^. This increases the memory voltage and lead to earlier filament formation^18, 23^. The comparison of the results in Fig. 10a,b relates the reduction of ozone concentrations to the excess power dissipation. At 18 and 20 kHz, current spikes led to saturation of the applied voltage. This made it difficult to control the amount of applied power and explains why the corresponding power values do not follow the linear trend with frequency. However, the most important information obtained from these pictures is that ozone rate follows a direct correlation with the average power consumption.

**Figure 11:**
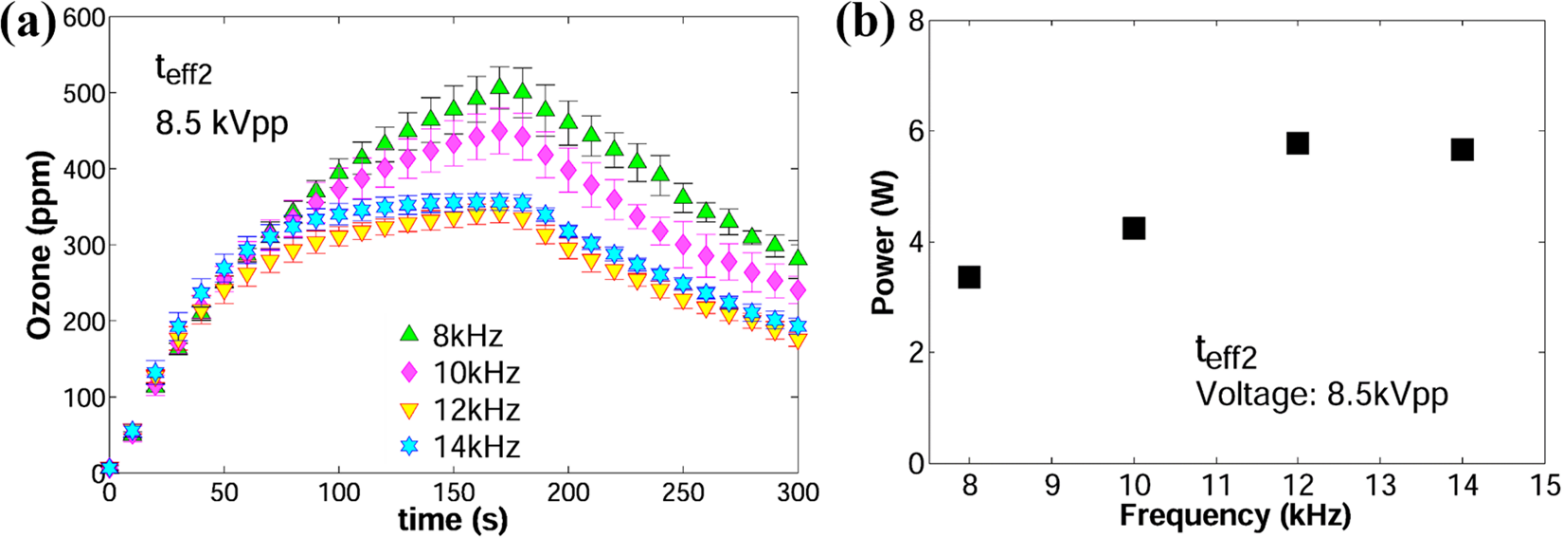
Curves of ozone vs. time in the frequency range 8–14 kHz. (**a**) Ozone rate vs. time. (**b**) Power vs frequency. Applied voltage = 8.5 kVpp; *t*_*eff*_ = 0.05 mm (*t*_*eff*2_).

To further explore the association between the acute destruction of ozone molecules and excess power dissipation, the same varying-frequency experiments were performed with *t*_*eff*2_ (0.05 mm). For this dielectric barrier, the applied voltage of 8.5 kVpp yielded excessive amount of power even at 8 kHz. Figure 11a shows that initial ozone levels for all frequencies reached a maximum or plateau followed by a fast decay, whose severity can be correlated to the power dissipation presented in Fig. 11b. At 12 kHz and 14 kHz, it was not possible to increment the applied voltage to 8.5 kVpp. Instead, the applied voltage dropped to 8.2 ~ 8.3 kVpp and the current levels became significantly high. This explains the similar average power and performance of ozone production. It is possible that in the presence of thermal filaments, current spikes lead to the saturation of the applied voltage *V*_*a*_ due to limitations of the power source. Another possible explanation is that leakage current becomes significant as the applied voltage approaches the breakdown limit of the dielectric; the reactor struggles to maintain the maximum power capacity and the applied voltage drops. After the reactor was turned off, the overall temperature of the chamber increased approximately by 1 °C for the cases of 8 kHz and 10 kHz, whereas for 12 kHz and 14 kHz the increment was about 2 °C.

Although there are known factors that lead to the development of leaders or filaments in surface DBD plasma such as frequency, applied voltage, pressure and shape of the exposed electrode; the exact processes that lead to the filamentary mode of surface DBD plasma are extremely complicated and not yet fully understood. For example, the signal shape of the applied voltage may influence the formation of leaders^14^. Also, there is compelling evidence to indicate that heat dissipation in the dielectric at extreme voltages can alter and induce the formation of filaments^24^. However, the formation of initial filaments usually occurs before the increase in average temperature of the plasma and dielectric barrier^18, 23^. Moreover, the internal temperature of these filaments, more than 1000 times higher than the bulk plasma temperature, develops in a microsecond scale while the increment of the average plasma temperature and the dielectric barrier is a gradual process that takes over several seconds^14, 24^. Although we do not dismiss the possible contribution of the dielectric temperature to filament formation and quenching of ozone in a large time-scale, its influence could not explain the ionization processes that extinguish ozone molecules in short time-scales. Therefore, more research is needed in this area for definitive conclusions.

## Conclusion

We have characterized the ozone production of DBD-plasma actuators in air and atmospheric pressure for quasi-uniform and early filamentary modes of DBD plasma. The results indicate that ozone production performance is inversely proportional to the logarithm of the effective dielectric thickness and does not follow directly the real physical thickness of the barrier. We reported this behavior under two different combinations of voltage and frequency (7 kV/10 kHz and 8.5 kV/14 kHz). The influence of the operating frequency over the ozone performance of DBD-plasma reactors was also explored. Experimental results showed that the initial ozone rate increases linearly with frequency. In this work, such increment follows a slope of 0.3 ppm. After approximately 60 seconds (*zone II*), the ozone rate maintained a constant value of 1.8 ppm/s for all frequencies. Moreover, instantaneous ozone levels that grow linearly with frequency do so at constant rate of about 16.5 ppm/Hz. In addition, power measurements also showed an inverse proportionality to the logarithm of the effective thickness and a linear increment with frequency. Furthermore, it was demonstrated that instantaneous ozone levels are directly proportional to power dissipation. To our knowledge, these reports that are critical to the study and design of DBD-plasma reactors have not yet been reported. Finally, we documented how excessive power levels related to the presence of prominent thermal filaments induced rapid ozone depletion for small effective thickness and high frequencies. Although this study was conducted specifically for surface DBD plasmas, we expect these results to be useful for other DBD configurations such as volume DBD. However, the ozone and power behavior reported here are strongly connected to memory effects characteristics of dielectric barrier discharges. Hence, more investigation is needed for other types of cold plasmas used in biomedical applications such as micro or nano-pulsed discharges, especially if the dielectric barrier is not used.

### Data Availability

The ozone and power data has been deposited in the figshare public repository (https://doi.org/10.6084/m9.figshare.5183452).
